# Varying High Levels of Faecal Carriage of Extended-Spectrum Beta-Lactamase Producing Enterobacteriaceae in Rural Villages in Shandong, China: Implications for Global Health

**DOI:** 10.1371/journal.pone.0113121

**Published:** 2014-11-18

**Authors:** Qiang Sun, Maria Tärnberg, Lingbo Zhao, Cecilia Stålsby Lundborg, Yanyan Song, Malin Grape, Maud Nilsson, Göran Tomson, Lennart E. Nilsson

**Affiliations:** 1 Center for Health Management and Policy, Key Lab of Health Economics and Policy Research of Ministry of Health in Shandong University, Shandong University, Jinan, Shandong, China; 2 Clinical Microbiology, Department of Clinical and Experimental Medicine, Linköping University, Linköping, Sweden; 3 Global Health (IHCAR), Department of Public Health Sciences, Karolinska Institutet, Stockholm, Sweden; 4 School of Public Health, Shandong University, Jinan, Shandong, China; 5 Antibiotics and Infection Control Unit, Public Health Agency of Sweden, Solna, Sweden; 6 Medical Management Centre (MMC), Department of Learning, Informatics Management, Ethics, Karolinska Institutet, Stockholm, Sweden; Cornell University, United States of America

## Abstract

Antibiotic resistance is considered a major threat to global health and is affected by many factors, of which antibiotic use is probably one of the more important. Other factors include hygiene, crowding and travel. The rapid resistance spread in Gram-negative bacteria, in particular extended-spectrum beta-lactamase (ESBL) producing Enterobacteriaceae (ESBL-E), is a global challenge, leading to increased mortality, morbidity and health systems costs worldwide. Knowledge about resistance in commensal flora is limited, including in China. Our aim was to establish the faecal carriage rates of ESBL-E and find its association with known and suspected risk factors in rural residents of all ages in three socio-economically different counties in the Shandong Province, China. Faecal samples and risk-factor information (questionnaire) were collected in 2012. ESBL-E carriage was screened using ChromID ESBL agar. Risk factors were analysed using standard statistical methods. Data from 1000 individuals from three counties and in total 18 villages showed a high and varying level of ESBL-E carriage. Overall, 42% were ESBL-E carriers. At county level the carriage rates were 49%, 45% and 31%, respectively, and when comparing individual villages (n = 18) the rate varied from 22% to 64%. The high level of ESBL-E carriage among rural residents in China is an indication of an exploding global challenge in the years to come as resistance spreads among bacteria and travels around the world with the movement of people and freight. A high carriage rate of ESBL-E increases the risk of infection with multi-resistant bacteria, and thus the need for usage of last resort antibiotics, such as carbapenems and colistin, in the treatment of common infections.

## Introduction

The faceless threat of antibiotic resistance is one of the greatest challenges of this century [1] and is now considered one of the three major threats to global health by the WHO. [2] Overuse and irrational use of antibiotics, both in human and veterinary medicine, are considered driving forces [3].

The rapidly increasing resistance in Gram-negative bacteria is a particularly serious problem worldwide, leading to increased mortality, morbidity and health systems costs. [2], [4], [5] The production of extended-spectrum beta-lactamases (ESBLs) in Enterobacteriaceae (ESBL-E) causes resistance to third-generation cephalosporins, one of the most important and widely used antibiotic classes. The co-acquisition of other resistance mechanisms is common, and multi-resistance is often seen, thus making treatment of infections with these bacteria difficult [6].

Overall, the knowledge about commensal flora and resistance is limited. Asymptomatic faecal carriage of ESBL-producing bacteria in the community has been reported from several countries and continents with wide differences in carriage rates between geographic areas and study population characteristics. Very high faecal prevalence rates have recently been reported from Thailand (66%), Egypt (63%) and China (50%). [7]–[9] In comparison, very low levels of carriage (3%) have recently been reported from Sweden [10].

Regarding faecal carriage of ESBL-E in China, few recent studies have been published. In western China a carriage rate of 39% was seen in 2006 to 2009 among hospitalized patients. [11] In 2012, a rate of 17% was found among healthy volunteers in Hangzhou, Zhejiang Province (eastern China). [12] When analysing different age categories the results vary. In an elderly population of Shenyang, Liaoning Province (north-eastern China), the carriage rate was only 7%. [13] In children admitted to one hospital in Hong Kong in 2007–8 the rate was 38%, and among their siblings it was 21%. [14] Finally, a 50% carriage rate was found in adults in Fuzhou, Fujian Province (eastern China), in 2009 [9].

The situation with respect to overuse of antibiotics and antibiotic resistance in China is very serious. [5] Several factors are interacting, including a health system with financial incentives for drug prescribing (including antibiotics). In 2009, China launched an ambitious health-care reform plan, aimed at providing basic health-care for all by 2020. It includes a “National Essential Medicines List”, to ensure the accessibility of essential drugs for all Chinese and to improve their rational use [15].

The new policy is now implemented throughout the different levels of administration, and covers both township health centres and village clinics in rural areas as well as community health centres in urban areas. To ensure accessibility, quality assurance and a feasible price for the end user, procurement has been implemented at the provincial level. Still, the rational use of medicine is an ongoing challenge at the primary care level in China. This prompted us to investigate faecal ESBL-carriage, in relation to antibiotic use and behaviours, in rural areas a few years after the health-care reform was presented, so as to further improve the rational use of essential medicines, with an emphasis on antibiotics.

The aim of this study was to establish the faecal carriage rates of ESBL-E and its association with several known and suspected risk factors, in rural residents of all ages in three socio-economically different counties in Shandong Province, China. To our knowledge this is the largest study of faecal carriage of ESBL-E performed in China.

## Materials and Methods

### Setting

**Figure 1:**
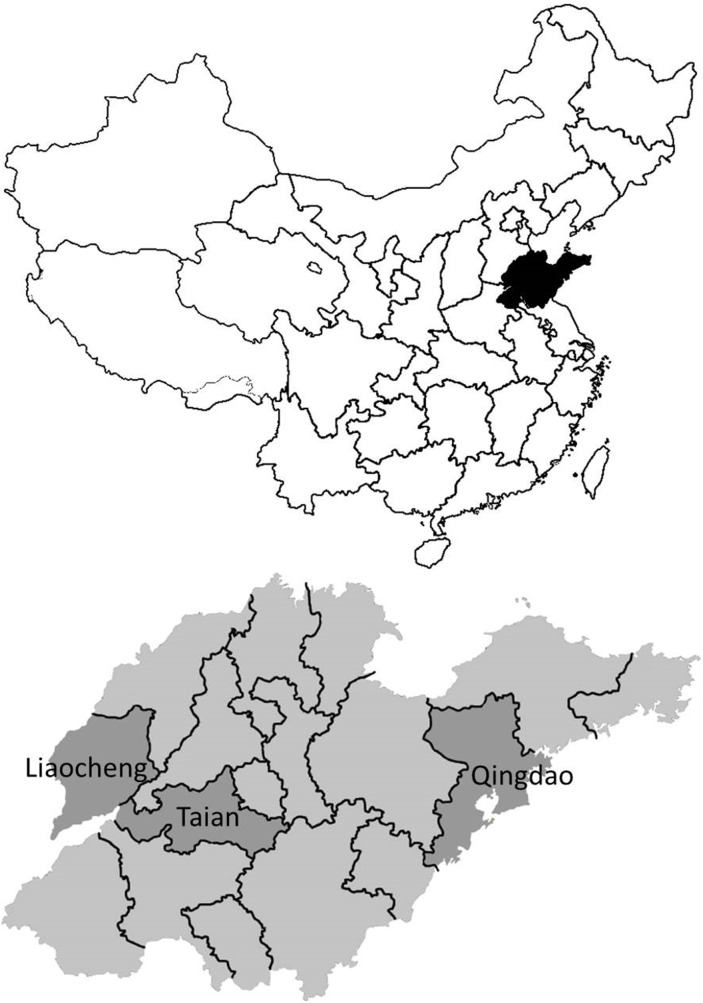
Map of China and Shandong.

The study was conducted in 2012 in Shandong Province, located by the coast in the eastern part of China, and covering an area of 157 000 sq.km. (Figure 1) The climate is temperate and typical crops grown in the area are wheat, corn, and sweet potato. Animal husbandry is intense and mainly consists of pig, cattle, chicken and goat farming. The population is around 97 million. The province of Shandong has a slightly higher income and better health indicators than China on average. The province is divided into 17 prefecture-level divisions and subdivided into 140 county-level divisions.

Rural life in China differs from rural life in the western hemisphere. Typically, a rural family in Shandong consists of two adults and one or two children living in a four- room house with a yard. A tap (or a well) and an outdoor toilet are located in the yard. Toilet paper is used and hand washing is commonly practiced. Toilet waste is usually disposed of by the family itself, and is sometimes used as crop fertilizer. Most families have hens, and many have pigs, and it is common to grow some vegetables in the yard.

The healthcare system in the rural areas of Shandong is organized at three different levels. There are modern, fully functional, county hospitals (including general hospitals and traditional Chinese hospitals). Below them are township health centres (similar to European primary care level), and lowest in the hierarchy are village clinics, with one or two doctors responsible for the whole village. In total, there were 1490 hospitals with almost 300 000 beds and 3 926 health centres in 2012 in the Shandong Province [16].

Three counties in different prefecture-level divisions were selected for this study, considering differences in economic development levels and geographic location (Table 1). Three townships in each county were randomly selected, within which two rural villages were subsequently randomly selected. Altogether, 18 villages were selected as study sites using multistage sampling based on the vertical administrative structure.

**Table 1:** Characteristics of the counties.

County	Prefecture-level division	Location	Population density	GDP/capita
			**(inh/sq.km)**	**(USD)**
**J**	Qingdao	east	453	12100
**N**	Taian	central	715	4600
**Y**	Liaocheng	west	747	3900

### Specimen collection and questionnaire survey

In this observational study the intention was to sample an even distribution among gender and age groups. Age groups were set to <7 years (preschoolers), 7–15 years (schoolchildren), 16–60 years (adults) and >60 years (elderly). The data collectors randomly sampled 60 rural residents in each village according to the roster provided by the village doctor. In total, 1080 residents were approached.

Faecal samples and questionnaires were collected in October 2012. A sample collection kit was given to each participant by the village doctor one day in advance, together with careful written, oral and pictorial instructions on handling. Participants (or parents of children under the age of 16 years) were interviewed individually, face-to-face using a standardized questionnaire. Questions were asked regarding socio-economic factors, living habits and medical behaviours (chronic diseases, hospitalization and drug use). Drug prescription data were obtained from the village doctors.

### Microbiological methods

Faecal samples were collected by the participants using a nylon flocked ESwab 480CE (Copan, Brescia, Italy). The swab was spread onto ChromID ESBL agar (BioMerieux, Marcy l’Etoile, France). To confirm an ESBL phenotype, combinatory Etests (BioMerieux) with a cephalosporin ± an inhibitor was used.

### Statistical analysis

Data were analysed using Stata/SE 13 software. Categorical data were compared using the X^2^ test, and univariate logistic regression analysis was used to explore risk factors associated with the prevalence of faecal ESBL-E. Significance was set at P<0·05. ESBL prevalence was investigated at all administrative levels (county, township and village); socio-economic factors, living habits and medical behaviours were investigated only at county level.

### Ethical approval

Ethical approval was granted by the Ethics Committee of the School of Public Health, Shandong University. Written consent forms were obtained from the participants (or parents of children under the age of 16 years) after appropriate information had been given.

## Results

### Study population

A total of 1 000 participants living in rural areas were included; 347 from J County, 315 from N County and 338 from Y County. For the age group 16–60 years, covering the working part of the population, the median age was 48 years, regardless of gender. Socio-economic factors, living habits and participants’ medical behaviour are further presented in Table S1. Non-participation was due to absence of questionnaire (n = 23), absence of sample (n = 12), inappropriate sampling (n = 2) or because the participant did not show up on the day of sampling (n = 43).

### Carriage of faecal ESBL-E

Overall, 42% of the participants carried faecal ESBL-E. No significant differences were seen regarding different socio-economic factors (Table 2). Among living habits (Table 3), “other source of water” was associated with ESBL carriage (OR 6·57, CI 1·40–30·7, P = 0·017), but in this small group of participants (n = 11) six stated their water source to be “pure water”, three “mineral water” and two “water from wells”.

**Table 2:** Socio-economic factors among participants.

	Total			County								
				J			N			Y		
	N = 1000			N = 347			N = 315			N = 338		
	ESBL+	OR	P	ESBL+	OR	P	ESBL+	OR	P	ESBL+	OR	P
	n (%)	(95% CI)		n (%)	(95% CI)		n (%)	(95% CI)		n (%)	(95% CI)	
**Total**	418 (42)			170 (49)	1.20	0.227	97 (31)	0.55	0.000*	151 (45)	1	
					(0.89–1.62)			(0.40–0.76)				
**Socio-economic factors**												
**Gender**												
***Male***	196 (41)	1		74 (47)	1		46 (30)	1		76 (45)	1	
***Female***	222 (43)	1.07	0.58	96 (50)	1.14	0.55	51 (31)	1.07	0.77	75 (44)	0.98	0.913
		(0.83–1.38)			(0.75–1.75)			0.66–1.73)			(0.64–1.50)	
**Age**												
***<7 years***	100 (45)	1		39 (61)	1		31 (40)	1		30 (37)	1	
***7–15 years***	107 (44)	0.97	0.889	47 (48)	0.59	0.107	17 (28)	0.57	0.131	43 (52)	1.87	0.049*
		(0.68–1.41)			(0.31–1.12)			(0.28–1.18)			(1.00–3.50)	
***16–60 years***	114 (38)	0.74	0.100	45 (43)	0.48	0.023*	30 (29)	0.59	0.100	39 (43)	1.28	0.437
		(0.52–1.06)			(0.26–0.91)			(0.32–1.11)			(0.69–2.35)	
***>60 years***	97 (41)	0.87	0.447	39 (49)	0.64	0.191	19 (26)	0.53	0.075	39 (46)	1.47	0.222
		(0.60–1.25)			(0.33–1.25)			(0.27–1.07)			(0.79–2.75)	
**Educational level**												
***16–60 years***												
**Illiterate**	24 (42)	1.57	0.212	11 (46)	1.92	0.232	7 (44)	1.44	0.593	6 (35)	1.27	0.732
		(0.77–3.22)			(0.66–5.61)			(0.37–5.57)			(0.32–5.06)	
**1–5 years**	24 (32)	1		11 (31)	1		7 (35)	1		6 (30)	1	
**>5 years**	66 (39)	1.40	0.249	23 (51)	2.38	0.065	16 (23)	0.56	0.292	27 (50)	2.33	0.129
		(0.79–2.49)			(0.95–5.96)			(0.19–1.64)			(0.78–6.98)	
***>60 years***												
**Illiterate**	60 (42)	0.93	0.829	30 (50)	1.25	0.719	13 (31)	1.07	0.907	17 (43)	0.63	0.368
		(0.50–1.74)			(0.37–4.15)			(0.31–3.69)			(0.23–1.71)	
**1–5 years**	21 (40)	1		6 (46)	1		1 (8)	1		14 (54)	1	
**>5 years**	16 (38)	0.57	0.205	3 (43)	0.88	0.888	5 (29)	0.20	0.169	8 (44)	0.69	0.540
		(0.24–1.36)			(0.14–5.56)			(0.02–1-98)			(0.20–2.29)	
**Annual household income (Yuan)**												
***<10 000***	93 (46)	1		33 (52)	1		19 (39)	1		41 (45)	1	
***10000–30 000***	218 (39)	0.91	0.520	83 (44)	0.93	0.765	56 (29)	0.76	0.319	79 (45)	1.07	0.786
		(0.68–1.22)			(0.56–1.53)			(0.44–1.31)			(0.66–1.73)	
***>30 000***	107 (44)	1.10	0.565	54 (56)	1.45	0.177	22 (29)	0.79	0.457	31 (44)	1.01	0.962
		(0.79–1.53)			(0.84–2.50)			(0.42–1.48)			(0.57–1.82)	

Note: Percentages are calculates on the total n for each county, and not within subgroupings. Statistically significant results are **bold** and marked with an *.

**Table 3:** Living habits among participants.

	Total			County								
				J			N			Y		
	N = 1000			N = 347			N = 315			N = 338		
	ESBL+	OR	P	ESBL+	OR	P	ESBL+	OR	P	ESBL+	OR	P
	n (%)	(95% CI)		n (%)	(95% CI)		n (%)	(95% CI)		n (%)	(95% CI)	
**Living habits**												
**Eating**												
***Non-vegetarians***	378 (42)	1		163 (49)	1		93 (32)	1		122 (44)	1	
***Vegetarians***	40 (39)	0.87	0.506	7 (54)	1.20	0.737	4 (15)	0.36	0.070	29 (46)	1.07	0.810
		(0.57–1.32)			(0.40–3.67)			(0.12–1.08)			(0.62–1.85)	
***Usually not eating raw vegetables***	300 (44)	1		107 (51)	1		81 (33)	1		112 (49)	1	
***Usually eating raw vegetables***	118 (38)	0.82	0.145	63 (46)	0.82	0.380	16 (24)	0.66	0.197	39 (36)	0.64	0.058
		(0.62–1.07)			(0.54–1.27)			(0.36–1.24)			(0.40–1.02)	
**Source of water**												
***Tap water***	226 (40)	1		72 (44)	1		35 (29)	1		118 (43)	1	
***Private well***	40 (50)	1.46	0.114	6 (43)	0.91	0.874	1 (17)	0.48	0.510	33 (55)	1.63	0.090
		(0.91–2.34)			(0.30–2.75)			(0.05–4.26)			(0.93–2.85)	
***Shared well***	144 (41)	1.01	0.965	84 (52)	1.30	0.243	60 (32)	1.13	0.644	-	-	-
		(0.77–1.32)			(0.84–2.00)			(0.68–1.85)				
***Other source of water***	9 (82)	6.57	0.017*	8 (100)	-	-	1 (50)	2.4	0.540	-	-	-
		(1.41–30.7)						(0.15–39.5)				
**Drinking**												
***Usually not drinking unbolied water***	387 (43)	1		162 (50)	1		97 (32)	1		128 (46)	1	
***Usually drinking unboiled water***	31 (33)	0.66	0.067	8 (38)	0.62	0.294	-	-	-	23 (37)	0.68	0.186
		(0.42–1.03)			(0.25–1.52)						(0.39–1.20)	
**Animals**												
***No pets in the house***	355 (42)	1		162 (50)	1		87 (30)	1		106 (44)	1	
***Pets in the house***	63 (43)	1.12	0.538	8 (33)	0.60	0.236	10 (40)	1.56	0.302	45 (46)	1.14	0.593
		(0.79–1.59)			(0.25–1.40)			(0.67–3.60)			(0.71–1.82)	
***No commercial farm nearby village***	294 (43)	1		34 (57)	1		13 (25)	1		77 (43)	1	
***Commercial farm nearby village***	124 (41)	1.01	0.971	136 (47)	0.70	0.210	84 (32)	1.45	0.281	74 (46)	1.25	0.300
		(0.76–1.33)			(0.40–1.22)			(0.74–2.86)			(0.82–1.93)	

Note: Percentages are calculates on the total n for each county, and not within subgroupings. Statistically significant results are **bold** and marked with an *.

Most factors exhibiting overall significance were found among medical behaviours (Table 4). Three of them were associated with drug use; i) recent intravenous injection (OR 1·52, CI 1·17–1·96, P = 0·001), ii) previous use of antibiotics (OR 1·56, CI 1·12–2·16, P = 0·008), and iii) discontinuous use of antibiotics (OR 1·36, CI 1·00–1·85, P = 0·046). Diabetes (n = 20) was also found to be associated with ESBL carriage (OR 4·27, CI 1·54–11·9, P = 0·005), but no details regarding type, treatment or severity were given.

**Table 4:** Medical behaviours among participants.

	Total			County								
				J			N			Y		
	N = 1000			N = 347			N = 315			N = 338		
	ESBL+	OR	P	ESBL+	OR	P	ESBL+	OR	P	ESBL+	OR	P
	n (%)	(95% CI)		n (%)	(95% CI)		n (%)	(95% CI)		n (%)	(95% CI)	
**Medical behavior**												
**Hospitalization**												
***Never hospitalized***	241 (41)	1		96 (52)	1		51 (27)	1		94 (44)	1	
***Ever hospitalized***	177 (43)	1.05	0.474	74 (46)	0.87	0.242	46 (37)	1.33	0.018*	57 (45)	1.00	0.968
		(0.92–1.20)			(0.70–1.10)			(1.05–1.67)			(0.79–1.26)	
***Hospitalised in 2012***	41 (42)	1.02	0.938	21 (47)	0.89	0.707	8 (33)	1.13	0.779	12 (43)	0.92	0.840
		(0.67–1.55)			(0.47–1.66)			(0.47–2.75)			(0.42–2.02)	
**Chronic disease**												
***Any chronic disease***	126 (45)	1.20	0.194	52 (51)	1.16	0.519	26 (30)	0.94	0.829	47 (52)	1.51	0.094
		(0.91–1.59)			(0.73–1.85)			(0.55–1.61)			(0.93–2.46)	
**≤** ***60 years***	55 (47)	1.25	0.272	22 (48)	0.94	0.85	13 (33)	1.07	0.86	20 (61)	2.16	0.04
		(0.84–1.85)			(0.50–1.78)			(0.51–2.21)			(1.02–4.56)	
***>60 years***	70 (43)	1.40	0.241	30 (54)	2.07	0.15	13 (27)	1.11	0.85	27 (47)	1.13	0.80
		(0.80–2.47)			(0.77–5.51)			(0.36–3.42)			(0.45–2.82)	
***Gastritis***	14 (39)	0.88	0.71	5 (50)	1.03	0.96	2 (15)	0.40	0.234	7 (54)	1.47	0.5
		(0.44–1.74)			(0.29–3.62)			(0.09–1.82)			(0.48–4-46)	
***Bronchitis***	17 (52)	1.49	0.258	7 (54)	1.21	0.737	1 (13)	0.31	0.282	9 (75)	3.89	0.045*
		(0.75–2.99)			(0.40–3.67)			(0.04–2.59)			(1.03–14.6)	
***Diabetes***	14 (70)	4.28	0.005*	6 (75)	7.47	0.061	2 (50)	2.27	0.415	6 (75)	3.83	0.103
		(1.54–11.9)			(0.91–61.4)			(0.32–16.4)			(0.76–19.2)	
**Medical treatments**												
***No intravenous injection in 2012***	225 (38)	1		77 (42)	1		61 (29)	1		87 (44)	1	
***Intravenous injection in 2012***	193 (48)	1.52	0.001*	93 (56)	1.81	0.007*	36 (36)	1.39	0.201	64 (46)	1.10	0.656
		(1.17–1.96)			(1.18–2.76)			(0.84–2.30)			(0.71–1.70)	
***Never used antibiotics***	65 (33)	1		26 (42)	1		14 (23)	1		25 (34)	1	
***Ever used antibiotics***	353 (44)	1.56	0.008*	144 (51)	1.32	0.320	83 (33)	1.63	0.142	126 (48)	1.79	0.034*
		(1.12–2.16)			(0.76–2.30)			(0.85–3.13)			(1.04–3.07)	
***Discontinuous use of antibiotics***	116 (49)	1.49	0.008*	49 (53)	1.20	0.442	23 (40)	1.63	0.108	44 (52)	1.51	0.102
		(1.11–1.99)			(0.75–1.94)			(0.90–2.94)			(0.92–2.48)	
***>1 antibiotic for the same illness***	83 (47)	1.28	0.14	33 (56)	1.38	0.26	13 (38)	1.45	0.32	37 (44)	0.97	0.89
		(0.92–1.77)			(0.78–2.42)			(0.69–3.03)			(0.59–1.04)	
***Self-adjusting the dose of antibiotics***	31 (39)	0.89	0.62	16 (48)	0.97	0.92	6 (25)	0.73	0.52	9 (41)	0.85	0.71
		(0.55–1.42)			(0.47–1.98)			(0.28–1.91)			(0.35–2.04)	
***Ending ab-treatment when symptoms disappear***	268 (44)	1.29	0.051	111 (50)	1.06	0.80	61 (33)	1.33	0.25	96 (48)	1.46	0.09
		(0.99–1.68)			(0.66–1.33)			(0.81–2.18)			(0.94–2.26)	

Note: Percentages are calculates on the total n for each county, and not within subgroupings. Statistically significant results are **bold** and marked with an *.

The significance of these figures disappeared when breaking down the data to county level. Recent intravenous injection was only significant in J County (OR 1·80, CI 1·18–2·76, P = 0·007) and previous use of antibiotics was only significant in Y County (OR 1·79, CI 1·04–3·07, P = 0·034). At the county level, hospitalization was found to be significant in N County (OR 1·33, CI 1·05–1·67, P = 0·018) and chronic bronchitis in Y County (OR 3·89, CI 1·03–14·6. P = 0·045), but the significance disappeared when merged with the total population.

Age showed significant differences at the county level, but disappeared when merged with the total population; in J County the age range 16–60 was less prone to ESBL-E carriage (OR 0·48, CI 0·25–0·91, P = 0·023) and in Y County the age range 7–15 was at higher risk (OR 1·87, CI 1·00–3·50, P = 0·049).

**Figure 2:**
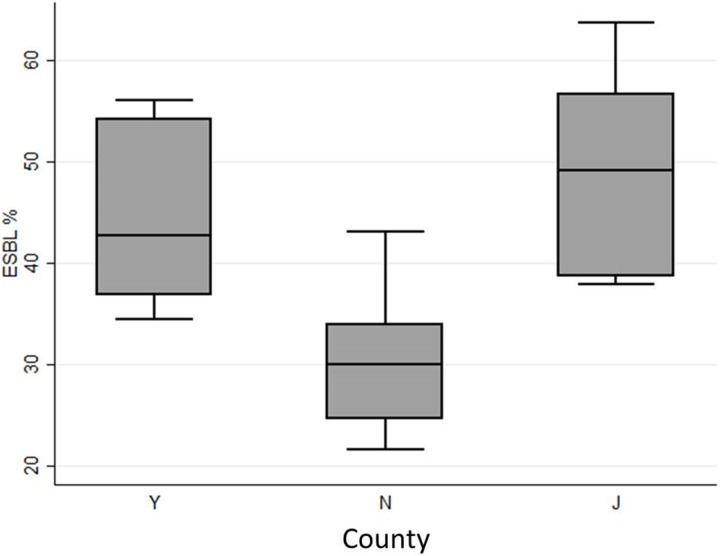
Box-and-whiskers-plot showing the differences in carriage-rate among the six villages in each county.

The prevalence was significantly different at all three levels investigated: county, township and village. In J County, 49% carried faecal ESBL-E, compared to 45% in Y County and only 31% in N County (OR 0·55, CI 0·40–0·76, P = 0·000). At township level (n = 9), the prevalence rate of ESBL-E varied from 28% to 52%, and when comparing individual villages (n = 18) the rate varied from 22% to 64% (Figure 2).

### Antibiotic prescriptions

Antibiotics accounted for 57–59% of all drug prescriptions in the three counties (40–69% when breaking down data to village level) in September 2012. The two most prescribed antibiotic classes were both broad spectrum, having an effect on Gram-negatives; third generation cephalosporins (mainly ceftriaxone), accounting for 20–28% of prescribed antibiotics; and quinolones, accounting for 11–16%. Carbapenems and colistin were not prescribed.

## Discussion

The main finding of this study is that although the level of faecal ESBL-E is high in rural Shandong (42%), it also varies greatly between villages (22–64%). We found no evident risk factors associated with this high carriage rate, but the statistical analysis indicates that the use of antibiotics may be one part of the explanation.

This is, to our knowledge, the largest study of faecal carriage of ESBL-E performed in China, both in terms of number of participants (n = 1 000), geographical distribution (18 measurement points at three different prefecture levels), and regarding analysis of potential risk factors. Altogether, previous studies involved a total of 787 participants (46–280 in each study), and only one of them investigated some risk factors. [9], [11]–[14] These studies presented a variation in carriage rate ranging from 7% to 50%, and all of them were performed in limited geographical areas.

There was an extremely high variation in faecal carriage rate of ESBL-E between the 18 investigated villages (22–64%), and the variation remained when data was merged to township (28–52%) and county level (31–49%). The risk factor analysis gave no clear answers as to why. These varying results highlights the risk of extrapolating data from limited point prevalence studies to larger areas.

Our overall risk factor analysis indicates that previous antibiotic use and intravenous injections are factors that may play a role regarding carriage of ESBL-E. “Intravenous injections” are often the same as intravenous antibiotics in rural China; therefore they can be used to get a rough indication of antibiotic use. Overuse of intravenous broad spectrum antibiotics for the “common cold” is a serious problem around the world, including China [17].

Previous use of antibiotics has been identified as a risk factor for community acquired UTIs caused by ESBL-E [18]–[20] but has infrequently also been found to be a risk factor for faecal carriage. Two reports, to our knowledge, have found a significant association between faecal carriage and antibiotic use; in elderly people in China (OR 3·2, CI 1·1–9·0, P = 0·03) [13] and in rural Thai communities (OR 1·88, CI 1·22–2·90, P = 0·004). [7] Others have found that antibiotic use was irrelevant for the faecal carriage of ESBL-E among healthy people [14], [21].

Cephalosporins and quinolones are regarded as the most potent ESBL-E selecting antibiotics. Prescriptions of cephalosporines and quinolones were proportionally high in all village clinics, regardless of county. We found no correlation between ESBL-E carriership and antibiotic prescription at either the village or county level. When this study was performed (October 2012) no carbapenems or colistin were prescribed in the investigated villages, but the risk of a high carriership of ESBL-E, manifesting itself as infections, may change this. The risk of overuse/misuse of these last resort drugs in the treatment of common, non-serious infections should not be underestimated.

High levels of antibiotic resistance, such as found in this study, have a multifactorial explanation. Antibiotic use in humans as well as animals is probably one of the most important factors, but also anthropogenic activities and lifestyle factors such as hygiene, crowding, and transportation etc. should be considered. When resistance is widely spread in a large area it is almost impossible to establish casual links – the resistance components have become a part of the residential flora.

The contribution of different sources to the current ESBL problem is difficult to assess, but intervention studies addressing the rational use of antibiotics may be implemented, since anthropogenic activities are easier to control than environmental factors. However, the outcome of such interventions is difficult to predict; previous interventions regarding for example vancomycin [22], [23] and trimethoprim-sulfa [24]–[26] did not manage to reverse the resistance levels.

Other types of study that are required in order to solve this ticking time bomb are:

- Intervention studies addressing adequate infection prevention and control
- Qualitative studies to understand, from the perspective of providers, patients and community, how antibiotic use can become more rational
- Molecular studies to better understand the complex dissemination routes of antibiotic resistance between humans, animals and the environment

This study gives robust evidence that antibiotic resistance is a severe problem among rural residents in China. Urgent and further implementation of the “Essential Medicines List”, with the priority on rational antibiotic use, is needed. Resistance knows no boundaries, and with further development of the Chinese economy, international transportation of people, freight, foodstuff and livestock to and from China will increase.

Containment of antibiotic resistance has been established as a “Global Public Good for Health”. [27] Improving consumer and provider interactions is essential for rational use of antibiotics [28] and improvements are necessary since antibiotics are not an endless resource. Although China is now taking important steps with its health care reform [15] there is also a need for global concerted actions [3] as well as a health systems perspective nationally involving communities, patients, health facilities, human resources, financial systems and inter-sectorial governance.

## Supporting Information

Table S1 **Socio-economic factors, living habits and participants’ medical behavior.**
(DOCX)
