# Supplementary material for: Varying High Levels of Faecal Carriage of Extended-Spectrum Beta-Lactamase Producing Enterobacteriaceae in Rural Villages in Shandong, China: Implications for Global Health
Source: PLoS One. 2014 Nov 18;9(11):e113121. doi: 10.1371/journal.pone.0113121 (PMC4236142; doi:10.1371/journal.pone.0113121)
Supplement: Table S1 — Socio-economic factors, living habits and participants’ medical behavior. (DOCX) [file pone.0113121.s001.docx]

**Table S1 Socio-economic factors, living habits and participants’ medical behaviour**

|  | Total | | County | | | | | |
| --- | --- | --- | --- | --- | --- | --- | --- | --- |
|  | **(n=1000)** | | **J (n=347)** | | **N (n=315)** | | **Y (n=338)** | |
|  | **Total** | | **Total J** | | **Total N** | | **Total Y** | |
|  | **n** | ***%*** | **n** | ***%*** | **n** | ***%*** | **n** | ***%*** |
| Total | 1000 | *100* | 347 | *100* | 315 | *100* | 338 | *100* |
| Socio-economic factors |  |  |  |  |  |  |  |  |
| Gender |  |  |  |  |  |  |  |  |
| *Male* | 478 | *48* | 156 | *45* | 153 | *49* | 169 | *50* |
| *Female* | 522 | *52* | 191 | *55* | 162 | *51* | 169 | *50* |
| Age |  |  |  |  |  |  |  |  |
| *<7 years* | 222 | *22* | 64 | *18* | 77 | *24* | 81 | *24* |
| *7-15 years* | 241 | *24* | 98 | *28* | 61 | *19* | 82 | *24* |
| *16-60 years* | 301 | *30* | 105 | *30* | 105 | *33* | 91 | *27* |
| *>60 years* | 236 | *24* | 80 | *23* | 72 | *23* | 84 | *25* |
| Educational level |  |  |  |  |  |  |  |  |
| *16-60 years* |  |  |  |  |  |  |  |  |
| Illiterate | 57 | *6* | 24 | *7* | 16 | *5* | 17 | *5* |
| 1-5 years | 76 | *8* | 36 | *10* | 20 | *6* | 20 | *6* |
| >5 years | 168 | *17* | 45 | *13* | 69 | *22* | 54 | *16* |
| *>60 years* |  |  |  |  |  |  |  |  |
| Illiterate | 142 | *14* | 60 | *17* | 42 | *13* | 40 | *12* |
| 1-5 years | 52 | *5* | 13 | *4* | 13 | *4* | 26 | *8* |
| >5 years | 42 | *4* | 7 | *2* | 17 | *5* | 18 | *5* |
| Annual household income (Yuan) |  |  |  |  |  |  |  |  |
| *<10 000* | 203 | *20* | 63 | *18* | 49 | *16* | 91 | *27* |
| *10 000-30 000* | 555 | *56* | 187 | *54* | 191 | *61* | 177 | *52* |
| *>30 000* | 242 | *24* | 97 | *28* | 75 | *24* | 70 | *21* |
| Living habits |  |  |  |  |  |  |  |  |
| Eating |  |  |  |  |  |  |  |  |
| *Vegetarians* | 103 | *10* | 13 | *4* | 27 | *9* | 63 | *19* |
| *Non-vegetarians* | 897 | *90* | 334 | *96* | 288 | *91* | 275 | *81* |
| *Usually eating raw vegetables* | 312 | *31* | 138 | *40* | 66 | *21* | 108 | *32* |
| *Usually not eating raw vegetables* | 688 | *69* | 209 | *60* | 249 | *79* | 230 | *68* |
| Source of water |  |  |  |  |  |  |  |  |
| *Tap water* | 556 | *56* | 162 | *47* | 119 | *38* | 275 | *81* |
| *Private well* | 80 | *8* | 14 | *4* | 6 | *2* | 60 | *18* |
| *Shared well* | 353 | *35* | 163 | *47* | 188 | *60* | 2 | *1* |
| *Other source of water* | 11 | *1* | 8 | *2* | 2 | *1* | 1 | *0* |
| Drinking |  |  |  |  |  |  |  |  |
| *Usually drinking unboiled water* | 94 | *9* | 21 | *6* | 11 | *3* | 62 | *18* |
| *Usually not drinking unbolied water* | 906 | *91* | 326 | *94* | 304 | *97* | 276 | *82* |
| Animals |  |  |  |  |  |  |  |  |
| *Pets in the house* | 146 | *15* | 24 | *7* | 25 | *8* | 97 | *29* |
| *No pets in the house* | 854 | *85* | 323 | *93* | 290 | *92* | 241 | *71* |
| *Commercial farm nearby village* | 709 | *71* | 287 | *83* | 262 | *83* | 160 | *47* |
| *No commercial farm nearby village* | 291 | *29* | 60 | *17* | 53 | *17* | 178 | *53* |
| Medical behaviours |  |  |  |  |  |  |  |  |
| Hospitalization |  |  |  |  |  |  |  |  |
| *Never hospitalized* | 590 | *59* | 186 | *54* | 192 | *61* | 212 | *63* |
| *Ever hospitalized* | 410 | *41* | 161 | *46* | 123 | *39* | 126 | *37* |
| *Hospitalized in 2012* | 97 | *10* | 45 | *13* | 24 | *8* | 28 | *8* |
| Chronic disease |  |  |  |  |  |  |  |  |
| *Any chronic disease* | 279 | *28* | 102 | *29* | 87 | *28* | 90 | *27* |
| ≤*60 years* | 118 | *12* | 46 | *13* | 39 | *12* | 33 | *10* |
| *>60 years* | 161 | *16* | 56 | *16* | 48 | *15* | 57 | *17* |
| *Gastritis* | 36 | *4* | 10 | *3* | 13 | *4* | 13 | *4* |
| *Bronchitis* | 33 | *3* | 13 | *4* | 8 | *3* | 12 | *4* |
| *Diabetes* | 20 | *2* | 8 | *2* | 4 | *1* | 8 | *2* |
| Medical treatments |  |  |  |  |  |  |  |  |
| *No intravenous injection in 2012* | 594 | *59* | 182 | *52* | 214 | *68* | 198 | *59* |
| *Intravenous injection in 2012* | 406 | *41* | 165 | *48* | 101 | *32* | 140 | *41* |
| *Never used antibiotics* | 197 | *20* | 62 | *18* | 61 | *19* | 74 | *22* |
| *Ever used antibiotics* | 803 | *80* | 285 | *82* | 254 | *81* | 264 | *78* |
| *Discontinuous use of antibiotics* | 235 | *24* | 93 | *27* | 58 | *18* | 84 | *25* |
| *>1 antibiotic for the same illness* | 177 | *18* | 59 | *17* | 34 | *11* | 84 | *25* |
| *Self-adjusting the dose of antibiotics* | 79 | *8* | 33 | *10* | 24 | *8* | 22 | *7* |
| *Ending ab-treatment when symptoms disappear* | 604 | *60* | 223 | *64* | 183 | *58* | 198 | *59* |
